# Quality Control in 3D Printing: Accuracy Analysis of 3D-Printed Models of Patient-Specific Anatomy

**DOI:** 10.3390/ma14041021

**Published:** 2021-02-21

**Authors:** Bernhard Dorweiler, Pia Elisabeth Baqué, Rayan Chaban, Ahmed Ghazy, Oroa Salem

**Affiliations:** 1 Department of Vascular Surgery, University Medical Center, Cologne 50937, Germany; oroa.salem@uk-koeln.de; 2 Department of Nuclear Medicine, University Medical Center, Johannes-Gutenberg University, 55131 Mainz, Germany; Pia-Elisabeth.Baque@unimedizin-mainz.de; 3 Department of Cardiothoracic and Vascular Surgery, University Medical Center, Johannes-Gutenberg University, 55131 Mainz, Germany; rayan.chaban@unimedizin-mainz.de (R.C.); ahmed.ghazy@unimedizin-mainz.de (A.G.)

**Keywords:** 3D printing, accuracy, 3D engineering, anatomical model, aorta, coronary, FDM printing, PolyJet printing

## Abstract

As comparative data on the precision of 3D-printed anatomical models are sparse, the aim of this study was to evaluate the accuracy of 3D-printed models of vascular anatomy generated by two commonly used printing technologies. Thirty-five 3D models of large (aortic, wall thickness of 2 mm, n = 30) and small (coronary, wall thickness of 1.25 mm, n = 5) vessels printed with fused deposition modeling (FDM) (rigid, n = 20) and PolyJet (flexible, n = 15) technology were subjected to high-resolution CT scans. From the resulting DICOM (Digital Imaging and Communications in Medicine) dataset, an STL file was generated and wall thickness as well as surface congruency were compared with the original STL file using dedicated 3D engineering software. The mean wall thickness for the large-scale aortic models was 2.11 µm (+5%), and 1.26 µm (+0.8%) for the coronary models, resulting in an overall mean wall thickness of +5% for all 35 3D models when compared to the original STL file. The mean surface deviation was found to be +120 µm for all models, with +100 µm for the aortic and +180 µm for the coronary 3D models, respectively. Both printing technologies were found to conform with the currently set standards of accuracy (<1 mm), demonstrating that accurate 3D models of large and small vessel anatomy can be generated by both FDM and PolyJet printing technology using rigid and flexible polymers.

## 1. Introduction

The “print revolution” was inaugurated when the technology of 3D printing (or additive manufacturing) transferred from industrial fabrication into science, where it opened new worlds to both researchers and clinicians [1].

Commonly used 3D printing technologies in the medical field include material extrusion with fused filament fabrication (FFF)/fused deposition modeling (FDM), vat photopolymerization with stereolithography (SLA) and material jetting (PolyJet), each of them with the ability to utilize either rigid or flexible polymers. While FDM employs a thermoplastic filament that is melted and extruded onto the build platform, both SLA and material jetting use liquid photopolymers that are either held in a vat or jetted onto the build platform and subsequently cured by ultraviolet light [2,3,4,5,6].

In cardiovascular medicine, current applications of 3D printing include the generation of patient-specific models of complex anatomy and disease for operative planning, training templates for education of students and residents and surgical templates for intraoperative navigation [7,8,9]. In addition, 3D-printed anatomical models were found to be advantageous in both preoperative patient communication and informed consent in complex aortic disease [10]. 3D printing technology also offers the possibility for cardiovascular bioprinting using biomaterials and living cells to create scaffolds or constructs for personalized medicine in cardiovascular disease and cardiovascular tissues (cardiac valves, epimyocardial patches, vascular conduits) [11]. Among the different applications for medical 3D printing mentioned above, production anatomical models (71%), followed by surgical guides and templates (25%), are currently dominating, whereas implants only account for <3% of prints [6,12]. In addition, 3D printing technology has been used to generate in vitro biomodels to assess the blood flow behavior in stenotic arteries and, consequently, to provide insights into both the cardiovascular disease condition [13] and dynamic 3D models that are used with a mock circulatory system to test and simulate cardiovascular functionality and hemodynamics [14]. With regard to their potential clinical impact, it could be demonstrated that the use of 3D-printed models in the preparation for endovascular procedures for aortic repair results in improvements in operative performance, like reduced operating time and reduced radiation exposure [5,7,15,16,17].

Despite their widespread use in clinical medicine, research and education, there is currently no standardized methodology for the generation of the 3D models, thus calling into question the reliability and accuracy of the constructs produced [18]. From the standpoint of quality control/assurance, with the use of existing 3D printing technologies and validated workflows, the process of printing 3D models can be performed with inaccuracies limited to less than typical image resolutions (<1 mm), but 3D-printed models should always be inspected against the designed STL model before they are used clinically [4,19]. An evaluation of the dimensional accuracy of 3D-printed models for cardiac applications was analyzed in a recent review [20]. Here, distance measurements were performed by caliper in the 3D models and compared to the original digital images, and inaccuracies in the range of 0.19–0.40 mm were noted [21,22,23]. However, those approaches were limited by the fact that a small number of anatomical hallmarks served as the reference for measurements and comparison. To overcome this drawback, computed tomography (CT)-scanning of the 3D models was employed in one study, thereby enabling direct measurement and comparison of digital images, which reported a spatial inaccuracy of 0.23 mm [20].

**Figure 1:**
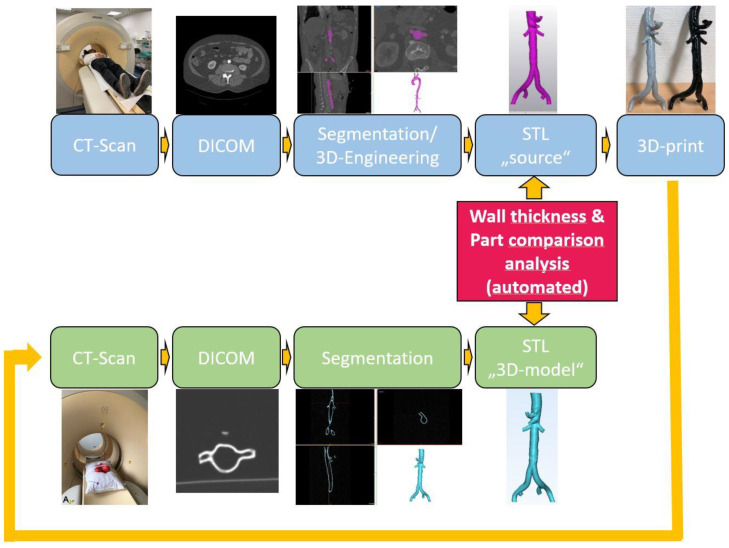
Schematic outline of study methodology: Standard workflow for in-house generation of 3D models is shown in the upper lane (light blue boxes). The specific workflow of this study is depicted in the lower lane (light green boxes). Here, the 3D-printed models were subjected to a high-resolution CT scan and an STL file was generated from the resulting DICOM dataset. Both STL files (source and 3D model) were then analyzed for wall thickness and three-dimensional part congruency using an automated software tool (Mimics Innovations Suite, Version 23, red box).

In our previous work, we used a specialized software solution for 3D engineering that provided advanced analysis tools, like surface comparison of whole parts, to discriminate conformational changes of endovascular aortic grafts [24]. Given that a high-resolution dataset of the 3D model was obtained, this method can be adopted to evaluate the surface congruency between two parts, namely, the 3D-printed model and the original dataset (STL file). Using this novel approach, the present study was designed to perform a head-to-head comparison of the “source” STL file and the STL file of the 3D-printed model, retrieved from a high-resolution CT scan, in order to evaluate a series of in-house produced medical 3D-printed models of patient-specific anatomy for their accuracy (Figure 1).

## 2. Materials and Methods

**Figure 2:**
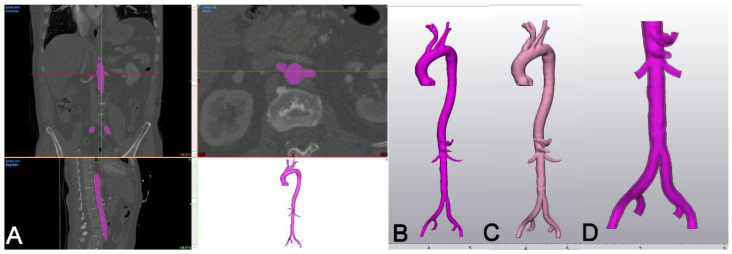
Generation of the original STL file (source STL). (**A**): With the Mimics Innovation Suite (MIS) software package, the blood volume (aorta and iliac bifurcation, fuchsia) was segmented from the original DICOM dataset (resolution 1 mm) and transformed into an STL file. (**B**): The STL file of the blood volume was then imported into the 3Matic module of MIS. (**C**): Using the “hollow” tool, a wall thickness of 2 mm was applied externally to the aorta. (**D**): Finally, the ostia were opened using the “trim” tool and the part of interest (abdominal aorta) of the model was exported as an STL file.

We established a full in-house workflow for 3D printing, and we currently use this technology for the generation of patient-specific models, training templates and patient education [25,26]. For the purpose of this study, a total of 35 consecutive 3D-printed models of patient-specific anatomy (large and small vessel), produced in rigid and flexible polymers, were selected. For the generation of the models, the anatomical structure of interest was segmented from a high-resolution contrast enhanced computed tomography angiography (CTA) with 1 mm sections. The DICOM dataset was then loaded into a dedicated 3D engineering software (Mimics Innovation Suite, Version 23, MIS, Materialise, Gilching, Germany). In case of aortic anatomy, the segmentation was performed using semi-automated “threshold” and “region growing” (26 connectivity) tools to delineate the intra-aortic blood volume. If needed, segmentation was refined by using the “multiple slice edit” tool (Figure 2A).

Next, the segmented object was exported as an STL file and loaded into the 3Matic module (Version 13) of MIS (Figure 2B), and a 2 mm wall thickness for aortic anatomy, or 1.25 mm thickness for coronary anatomy, was applied to the segmented blood volume using the “hollow” tool (Figure 2C). Finally, the ostia of the aorta and the branch vessels were opened using the “trim” tool (Figure 2D), and the final object was converted into an STL file, serving as the source STL for printing. Two different 3D printing technologies were used: first, fused deposition modeling (FDM) printing, which was performed by an Ultimaker II/III printer (nozzle 0.4 mm) using a standard polylactic acid (PLA) filament (diameter 2.85 mm, melting point 145–160 °C, density 1.24 g/cm^3^, flexural strength 103 MPa, hardness (Shore D) 83, manufactured by Ultimaker B.V., Watermolenweg 2, 4191 PN, Geldermalsen, The Netherlands). The STL source file was converted into a G-code using the proprietary Cura Software Version 4.8 (quality setting: high). In addition, a PolyJet technology (Stratasys Object 30 Prime) was employed to generate flexible 3D models using polymers of the Tango^®^ series (liquid photopolymer with proprietary composition, polymerized by UV light-curing, density (polymerized) 1.16 g/cm^3^, tensile strength 3–5 MPa, hardness (Shore A) 73–77, manufactured by Stratasys, 1 Holtzman St., Science Park, Rehovot, Israel). For both 3D printing technologies, the respective printing resolution was set to maximum with a layer resolution of 20–200 µm (Ultimaker II/III) and 28 µm (Stratasys Object 30 Prime), respectively. A thorough cleaning/removal of the support of the models was performed post-printing.

**Figure 3:**
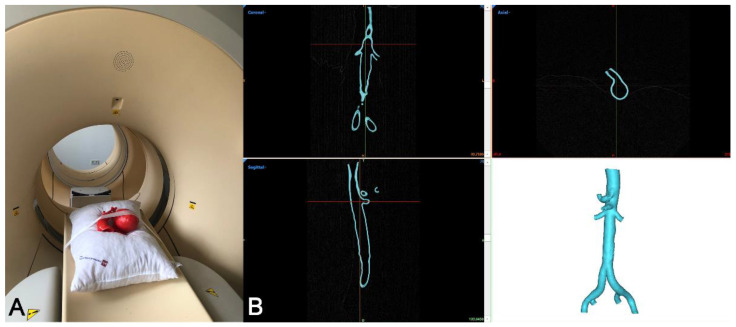
CT scanning of the 3D-printed model. (**A**): A high-resolution CT scan (slice thickness 0.4 mm) was obtained from all 35 3D-printed models. (**B**): The 3D model was segmented using MIS, and an STL file (3D model STL) was generated.

In order to perform an accuracy analysis of the 3D-printed models, they were subjected to high-resolution CT scanning [19]. In brief, the models were placed on a soft polyester cushion, and then a high-resolution helical CT scan with a pitch of 0.313, 90 kV, mAS/slice 74, pixel size 0.26 × 0.26 mm^2^, a slice thickness of 0.4 mm and collimation of 16 × 0.75, was performed (Figure 3A). The dataset obtained (DICOM files) was then segmented and converted into an STL file (Figure 3B) using the Mimics Innovation Suite software, as described above. For the purpose of this work, the latter STL file is referred to as the “3D model” STL.

Both the source STL and the 3D model STL files were then subjected to an accuracy/deviation analysis, as described earlier [24].

**Figure 4:**
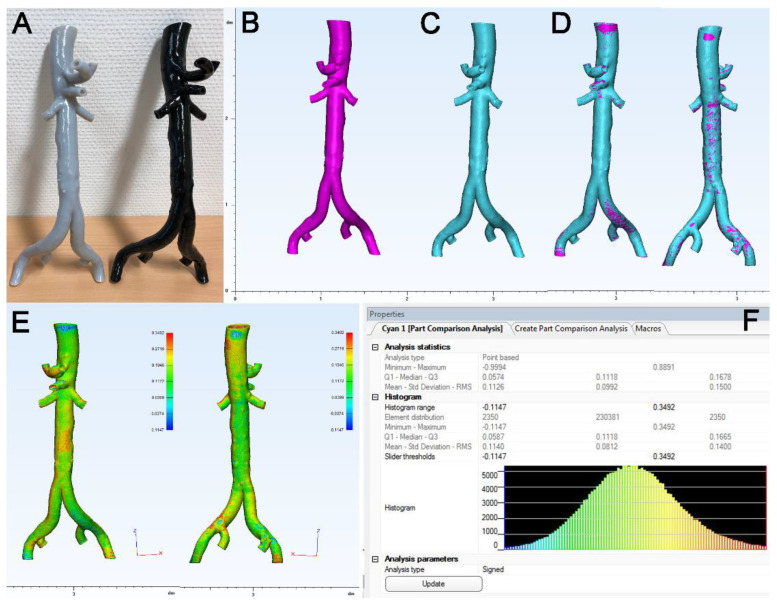
Analysis of surface congruency of 3D model STL versus source STL. (**A**): Two representative models of abdominal aorta printed in flexible and rigid polymer. (**B**): The source STL (fuchsia) and (**C**): the STL of the 3D model (cyan) were imported in the 3Matic module of MIS and (**D**): both objects were brought to congruency using semi-automated alignment tools. (**E**): The part comparison analysis tool of the 3Matic shows a color-coded map of the object indicating the surface deviation between both objects in mm. (**F**): Histogram report of the results indicating the mean value of surface deviation.

For the analysis of the surface accuracy/deviation of the 3D-printed models (Figure 4A), both objects (3D model, fuchsia, Figure 4B; source file, cyan, Figure 4C) were first aligned using the semi-automatic “interactive translate” and “global registration” tools (Figure 4D). Then, the part comparison analysis (PCA) tool was used to assess the distance (deviation) of surface triangles between the source STL and the 3D model STL (entity: 3D model STL, target: source STL). The results of this analysis are given as a color-coding of the 3D model (Figure 4E) and a respective histogram (Figure 4F). For further statistical analysis, the mean value given in the histogram was used.

**Figure 5:**
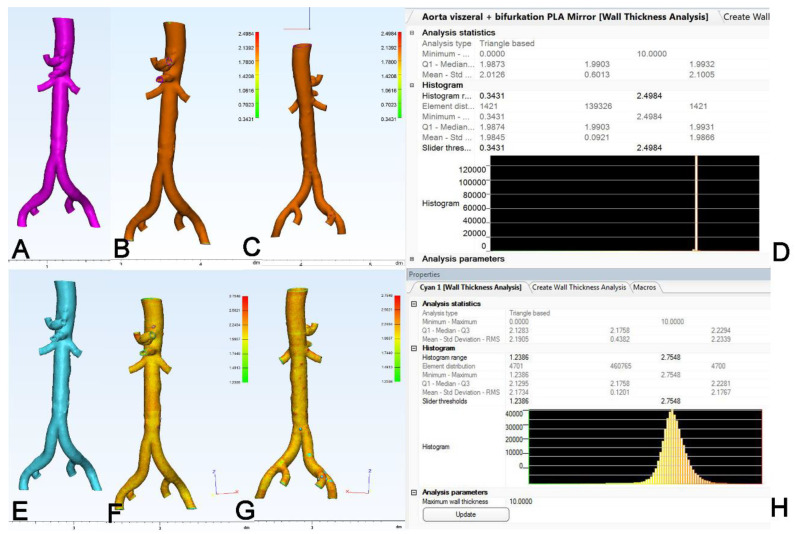
Analysis of wall thickness of the 3D model. For internal validation, (**A**): the source STL was subjected to automated analysis of wall thickness. (**B**,**C**): Results of wall thickness analysis as a color-coded map of the object where (**B**): is the anterior view and (**C**): is the posterior view. (**D**): Histogram of wall thickness analysis (WTA) showing a sharp band at 1.99 mm. For the STL of the (**E**): 3D model, the results of WTA are shown as a color-coded map in (**F**): anterior view, (**G**): posterior view and (**H**): histogram.

In addition, the wall thickness analysis (WTA) tool was employed to check the wall thickness of the 3D-printed model (Figure 5A). For internal validation of the thickness analysis, the source STL was also tested: here, the WTA results, given as a color-coded map (Figure 5B,C) and a histogram (Figure 5D), demonstrated an exact match for wall thickness with the value of 2 mm that was chosen when the model was designed. For the 3D model (Figure 5E), the results of the WTA are again given as a color-coding of the 3D model (Figure 5F,G) and a histogram (Figure 5H), and for further statistical analysis, the mean thickness was used. Of note, during the generation of the source STL file for the models of aortic anatomy, the wall thickness was set to 2 mm for the aortic models and 1.25 mm for the coronary models.

Statistical analysis was performed with SPSS software (SPSS 23.0 for Windows) and the mean and standard deviation were calculated. For statistical comparison between groups, the Mann-Whitney test was used for comparison of the non-Gaussian distribution (PCA), and the paired t-test was used for comparison of the wall thickness analysis of the 3D model STL vs. the given wall thickness of 2/1.25 mm. A *p* < 0.05 was considered significant, and a *p* < 0.01 was considered highly significant.

## 3. Results

In total, 35 3D models of patient-specific anatomy, including 20 models using FDM printing technology and 15 models using PolyJet technology, were engineered, printed and evaluated for printing accuracy. The respective anatomical structures were normal aortic anatomy (n = 10), coronary artery anatomy (n = 5), aortic aneurysm (n = 12) and aortic dissection (n = 8).

**Figure 6:**
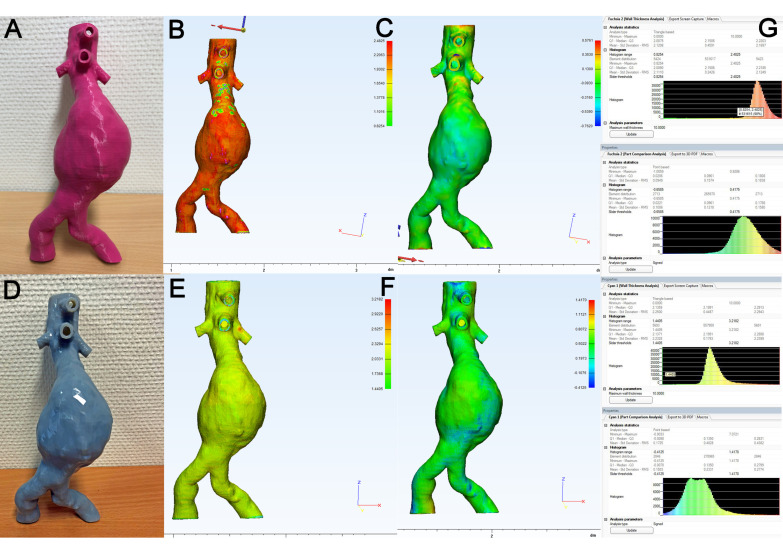
Representative examination of 3D model of infrarenal aortic aneurysm. (**A**): 3D model generated in fused deposition modeling (FDM) printing (rigid polylactic acid (PLA)). (**B**): Color map of WTA. (**C**): Color map of part comparison analysis (PCA). (**D**): 3D model generated in PolyJet printing (flexible polymer, Stratasys Tango^®^ gray, Rehovot, Israel). (**E**): Color map of WTA. (**F**): Color map of PCA. (**G**): Histogram results of WTA (lower middle panel) and PCA (lower panel).

The representative evaluation results of the 3D models of aortic aneurysm printed in FDM (Figure 6A) and PolyJet technology (Figure 6D) are shown in Figure 6. The WTA (Figure 6B) showed an overall uniform result of the FDM model with some minor spots (yellow/greenish) of reduced wall thickness in the aneurysm neck. The corresponding mean wall thickness was 2.11 mm (Figure 6G, upper panel). The PCA (Figure 6C) results demonstrated an overall uniform result (green) with some minor hotspots of increased deviation between the source file and the 3D model in the visceral part of the aorta (branch vessels). The corresponding mean value for PCA was 0.100 mm (Figure 6G, upper middle panel). Regarding the PolyJet model, the results of the WTA are shown in Figure 6E: Here, a uniform color distribution was visible with some minor hotspots at the branch vessel (left renal artery), and the mean value of wall thickness was 2.23 mm (Figure 6G, lower middle panel). The PCA (Figure 6F) showed a uniform color-coding (green) with a mean deviation of 0.15 mm (Figure 6G, lower panel).

**Figure 7:**
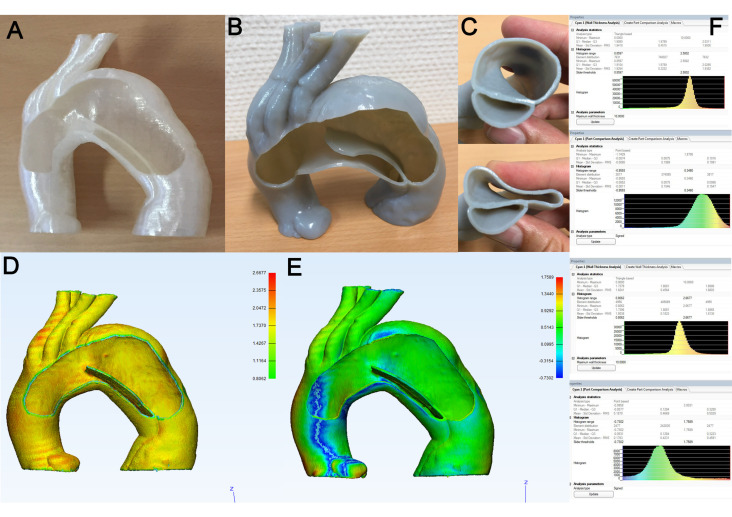
Representative examination of 3D model of aortic dissection. (**A**): 3D model generated in FDM printing (rigid PLA). (**B**): 3D model generated in PolyJet printing (flexible polymer, Stratasys Tango^®^ gray). (**C**): Flexibility of the model is shown by digital compression. (**D**): Color map of WTA. (**E**): Color map of PCA. (**F**): Histogram results of WTA (upper panel) and PCA (upper middle panel) for model A, and histogram results of WTA (lower middle panel) and PCA (lower panel) for model B.

For the models of aortic dissection, the representative parts are shown in Figure 7: The FDM-printed model (Figure 7A) demonstrated a mean wall thickness of 1.92 mm (Figure 7F, upper panel) and a mean deviation of the 3D model STL versus the source STL in the PCA of 0.001 mm (Figure 7F, upper middle panel). The flexibility of the PolyJet-printed model (polymer: Stratasys Tango^®^ gray, Shore hardness 75) of aortic arch dissection (Figure 7B) can be assessed in Figure 7C (upper and lower panels). The WTA (Figure 7D) showed overall satisfactory results with some minor areas of increased thickness (supra-aortic branch vessels), as well as reduced wall thickness (cutting edge of the window), and a mean WTA of 1.80 mm (Figure 6F, upper middle panel). The PCA (Figure 7E) demonstrated good overall congruency of the 3D-printed model compared to the source STL, with some minor deviation (blue) in the ascending aorta. The mean deviation between the 3D model STL and the source STL was 0.18 mm (Figure 7F, lower panel).

**Figure 8:**
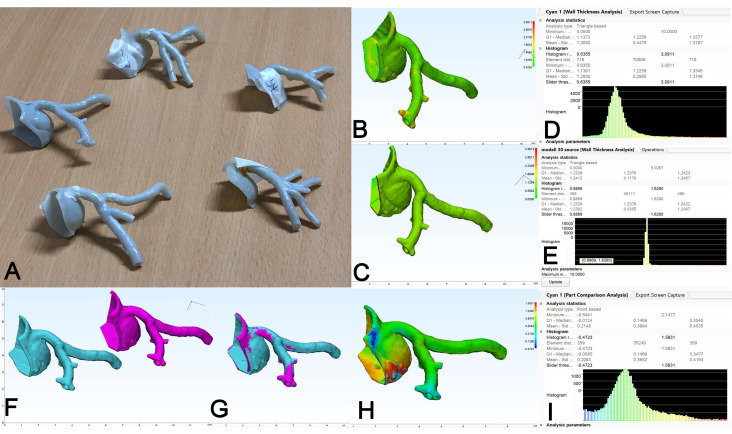
Representative examination of 3D model of coronary arteries. (**A**): 3D models of five different configurations of coronary arteries were generated and printed in flexible polymer (Stratasys Tango^®^ gray). (**B**): 3D model STL (cyan) and (**C**): source STL (fuchsia). (**D**): Color-coded map of WTA and histogram results of 3D model STL are shown. (**E**): Color map and histogram with sharp band of WTA of source STL for internal validation. (**F**): Source STL (fuchsia) and 3D model STL (cyan) were then semi-automatically aligned and overlayed (**G**). (**H**): Color map and (**I**): histogram results of PCA. Note surface deviation (hotspot) in yellow-red coding at coronary sinus.

In order to assess the printing accuracy in small vessel anatomy, five 3D models of different forms of coronary artery anatomy were printed using PolyJet technology (Tango^®^ gray) (Figure 8A). Here, the WTA yielded a uniform result in the color-coded image (Figure 8B) and a mean thickness of 1.28 mm in this representative case (Figure 8D). Internal validation of the WTA measurement for the small vessel models was assured again using the source STL (Figure 8C,E). As mentioned above, due to the flexibility of the coronary models, deformation can occur during the post-printing storage of the models and/or the fixation during the CT scan. When source STL (Figure 8F, fuchsia) and 3D model STL (Figure 8F, cyan) were overlayed (Figure 8G), the PCA yielded a mean deviation of 0.21 mm (Figure 8I) and demonstrated good congruency of both parts at the coronary bifurcation (Figure 8H), whereas substantial deviation can be noted at the base of the model (aortic anulus).

For overall comparison of wall thickness for all models, the mean WTA values obtained were normalized to the original wall thickness of the source STL (2 mm for aortic and 1.25 mm for coronary anatomy), and the overall analysis of the 35 models showed a mean relative wall thickness +5% (±9%). The overall value for surface congruency/deviation of all 35 models obtained in PCA was +0.12 mm (±0.08 mm). For further evaluation of wall thickness, the aortic and coronary models were analyzed separately: Here, a mean wall thickness of 2.11 mm (±0.19 mm) was noted for all aortic models with a respective mean thickness of 2.04 mm (±0.18 mm) for the FDM-printed and 2.26 mm (±0.51 mm) for the PolyJet-printed aortic models. The mean wall thickness of the coronary models (all printed in PolyJet technology) was 1.26 mm (±0.05 mm). Statistical analysis revealed no significant difference of FDM-printed aortic models and coronary models versus the source STL (*p* = 0.42 and *p* = 0.55, respectively); however, the aortic models printed in PolyJet technology showed a significant increase in mean wall thickness (*p* = 0.001). With regard to surface congruency, the mean deviation obtained in PCA was 0.10 ± 0.08 mm for aortic models and 0.18 ± 0.01 mm for coronary models (*p* = 0.004). Again, we found a significant difference in the mean values for surface congruency obtained in PCA with 0.09 ± 0.09 mm and 0.15 ± 0.03 mm for FDM and PolyJet technology, respectively (*p* = 0.01). An in-depth analysis on the individual color-coded images of WTA and PCA revealed that the difference noted between the two printing technologies predominantly resulted from either the surface deviations that were induced during storage, the positioning of the models for the CT scan or the remainders of support material within the models.

**Figure 9:**
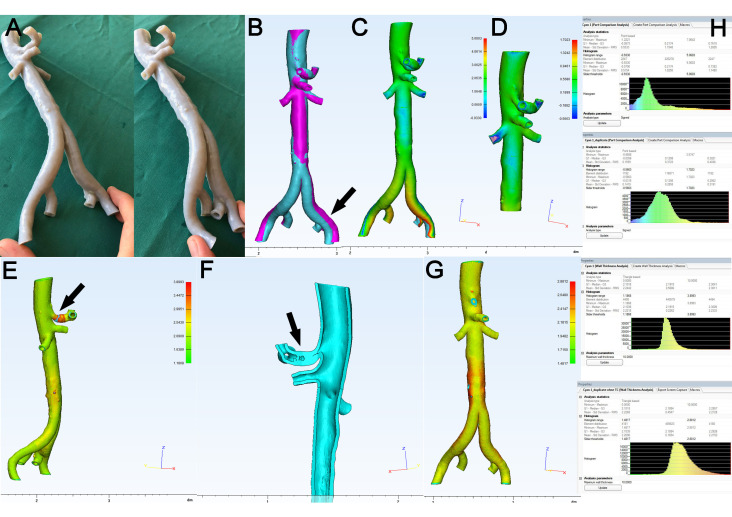
Representative depiction of artifact analysis. (**A**): Demonstration of flexibility of 3D model of abdominal aorta (Stratasys Tango^®^ gray). (**B**): Overlay of source STL (fuchsia) and 3D model STL (cyan) shows considerable surface deviation at left iliac axis (arrow). (**C**): PCA confirms hotspot at left iliac axis and mean deviation of 0.51 mm ((**H**), upper panel). (**D**): After removal of distal aorta, PCA shows uniform color map and mean deviation decreased to 0.14 mm ((**H**), upper middle panel). (**E**): Color map of WTA of abdominal aorta shows hotspot at the celiac trunk (arrow) caused by intraluminal remainders of support material in this hard-to-reach area, (**F**): anterior-posterior section (arrow). (**G**): For testing purposes, the celiac trunk was removed from the model and (**H**): mean wall thickness decreased from 2.22 to 2.20 mm (lower middle and lower panels).

Figure 9 depicts a representative example for both situations: As shown in Figure 9A, deformation of the geometry of aortic bifurcation can easily be induced by manual compression and storage/fixation of the model, which in turn resulted in a deviation of the 3D model STL (in cyan) when overlayed onto the source STL (in fuchsia) (Figure 9B, black arrow). Consequently, the PCA depicted a hotspot at the left iliac axis (Figure 9C) and a mean value of deviation of 0.51 mm (Figure 9H, upper panel). When the model was cut at the level of the distal aorta and the remaining piece (Figure 9D) was subjected to PCA again, a mean value of only 0.14 mm of surface deviation was noted (Figure 9H, upper middle panel). The color-coded images of WTA allow for the identification of so called hotspots with increased wall thickness (Figure 9E, black arrow). In this case, the model was cut in an anterior-posterior plane and remainders of the support material could be identified in the proximal part of the celiac trunk (Figure 9F, black arrow). Again, when the celiac trunk was cut (removed) from the model, this resulted in a reduced mean thickness of 2.20 mm of the remaining aortic part, compared to 2.22 mm of the original model (Figure 9H, lower middle and lower panels). Of note, both segmental analyses shown were only done for explanatory reasons while for the purpose of statistical analysis in this study, only measurements of the whole models were included.

## 4. Discussion

The overall analysis of the 35 3D prints demonstrated a relative increase of 5% in wall thickness compared to the source STL, which is in line with studies on aortic and kidney models that reported differences between the print and original STL files of up to 7% [27] and up to 8.6%, respectively [28]. In the subgroup analysis, it was shown that the printing precision in large (aortic) and small (coronary) vessel anatomy was excellent, with a mean wall thickness of +0.04 mm for FDM-printed aortic and +0.01 mm for coronary models, and no significant difference compared to the source STL file (2 and 1.25 mm, respectively), with *p* = 0.42 and *p* = 0.55, respectively. However, regarding the printing precision of the PolyJet-printed aortic models, a significant increase in mean wall thickness of +0.26 mm was evident (*p* = 0.001), which could be attributed to pieces of the support material that remained within the models in hard-to-reach areas (small vessel branches), despite both water jet and ultrasound cleaning of the models. However, even under these circumstances, and taking into account the spatial inaccuracy of CT scanning of ±200 µm (for 0.4 mm slices), a printing accuracy of +260 µm for PolyJet printing demonstrated a result that was well within the accuracy limits (<1 mm) that are currently postulated [4,19,29].

The novel contribution of the current study is the introduction of a dedicated 3D engineering tool (part comparison analysis) that enables an automated high-precision three-dimensional analysis of surface geometry and congruency between two parts. Applying this tool, we were able to show that the respective mean surface deviation was +100 µm for aortic and +180 µmm for coronary 3D models. For FDM-printed models, we noted a mean deviation of as low as +90 µm compared to the source STL file. Similar to the wall thickness analysis, we again found a significant increase of +150 µm (*p* = 0.01) in the mean surface deviation for the PolyJet-printed aortic models, and we could identify post-printing deformation/bending of the models that were minor in all cases and resulted from the increased flexibility of the PolyJet-printed models. However, it should be stressed again that a printing precision with a mean surface deviation of +150 µm is still in line with current recommendations for printing accuracy [4,19]. A high printing accuracy, in turn, enables physicians to use 3D-printed anatomical models in the context of clinical applications, for instance, in the generation of surgeon-modified endovascular stent-grafts for the treatment of complex aortic pathologies [30,31,32]. Potential limitations of our study include the fact that it was designed to assess the accuracy of our self-established in-house 3D printing workflow, and consequently, we cannot provide data at this stage on the clinical impact of those models. This, however, will be the focus of a follow-up study. Notably, depending on the intended use of 3D-printed models, the requirements of the current EU Regulation on Medical Devices 2017/745 (known as MDR) will apply to the 3D printing process as long as clinical applications are intended.

## 5. Conclusions

We focused on two main criteria for printing accuracy, namely, the wall thickness of the hollow models and the surface congruency of the prints compared to the source STL file, and we took advantage of a specialized 3D engineering software (MIS) for the analysis of both parameters. With this approach, we were able to show that models of patient-specific anatomy can be generated using 3D printing technology, with high accuracy using both FDM and PolyJet technology. The potential advantages of FDM technology are the relatively low costs of both the printer and printing polymers when compared to PolyJet technology. However, limitations of FDM technology arise with increasing complexity and larger dimensions (>30 cm) of the models, multi-color printing and the generation of flexible or transparent objects. PolyJet technology, in turn, offers the use of flexible and transparent polymers, and the current generation of PolyJet 3D printers enables multi-color and even multi-material (flexible and rigid) printing processes in large scale (>30 cm), albeit at higher costs for both the printer and polymers. Therefore, depending on the intended application of anatomical 3D models, a 3D printing lab should be equipped with different printing technologies to accommodate the requirements. 3D printing will play an increasingly important role in enabling patient-oriented precision medicine [19], and future advances regarding software solutions, printing polymers and easy-to-handle printers will further propagate and expand the applicability of 3D printing technology in cardiovascular medicine.

## Data Availability

Data sharing is not applicable to this article.
